# Talc Exposure and Risk of Ovarian Cancer: A Systematic Review and Meta-Analysis with Limited Evidence on Cervical and Endometrial Cancers

**DOI:** 10.3390/cancers18101589

**Published:** 2026-05-13

**Authors:** Monireh Sadat Seyyedsalehi, William Anthony Powley, Paolo Boffetta

**Affiliations:** 1 Department of Medical and Surgical Sciences, University of Bologna, Via Massarenti 9, 40138 Bologna, Italy; monireh.seyydsalehi@unibo.it; 2 Formidable Partners, Boston, MA 01741, USA; wpowley@formidable.partners; 3 Stony Brook Cancer Center, Stony Brook University, Stony Brook, NY 11794, USA; 4 Department of Family, Population and Preventive Medicine, Renaissance School of Medicine, Stony Brook University, Stony Brook, NY 11794, USA

**Keywords:** talc, genital exposure, malignant, ovary, endometrium, fertility

## Abstract

**Introduction:** Talc, commonly used in products like baby powder and cosmetics, has been studied for a possible link to female genital cancers, especially ovarian cancer. However, evidence is inconclusive. We conducted a systematic review and meta-analysis to assess the association between talc exposure and the incidence and mortality of ovarian, cervical, and endometrial cancers, including ovarian cancer subtypes. **Method:** We searched MEDLINE (PubMed) and SCOPUS databases up to January 2026 for cohort and case–control studies. Studies were assessed for quality using a modified Newcastle–Ottawa Scale. Due to the nature of available data, meta-analysis was performed only on case–control studies on ovarian cancer, using a random-effects model. Publication bias was assessed through funnel plots and Egger’s test, and a leave-one-out sensitivity analysis was performed. Stratified analyses were conducted based on geographic location, year of publication, exposure source, and tumor characteristics. Additionally, a two-stage dose–response meta-analysis was performed for ovarian cancer regarding frequency and duration of talc use. **Results:** Our review identified 37 studies related to ovarian cancer, one to cervical cancer, and six to endometrial cancer. Twenty-five case–control studies were included in the meta-analysis. Results showed a positive association between talc use and ovarian cancer risk (RR 1.32; 95% CI: 1.25–1.39), with stronger associations observed for women who applied talc directly to the genital area (RR 1.38) and those who used talc after bathing (RR 1.30). The risk was also higher for specific ovarian cancer subtypes, such as serous (RR 1.36) and endometrioid tumors (RR 1.35). No evidence of publication bias was found. Results of the pooled analysis of four cohort studies showed no association (RR for ever-use 1.08; 95% CI: 0.99–1.17, no trend according to duration or frequency of use). For cervical and endometrial cancers, no significant associations with talc exposure were observed. **Conclusions:** Case–control studies suggest a modest link between genital talc use and ovarian cancer risk, especially serous and endometrioid types. Cohort studies show no association, and no links were found for cervical or endometrial cancers. Accordingly, the results should be interpreted with caution, and the current evidence does not conclusively establish a causal relationship.

## Simple Summary

Talc is a mineral commonly used in personal care products such as baby powder and cosmetics, and many women have used it on or near the genital area. There has been debate about whether this type of talc use might increase the risk of cancers of the female reproductive organs, especially the ovary. To help clarify this issue, we systematically reviewed and first combined results from cohort and case–control studies where possible to provide an overall estimate of risk and then examined results separately by study design for ovarian, cervical, and endometrial cancers. We found that case–control studies showed a positive association for ovarian cancer, particularly with frequent or long-term genital use, supported by leave-one-out sensitivity analyses, while long-term follow-up cohort studies did not show an association. For endometrial and cervical cancers, no significant associations were observed, although the number of available studies was limited. These findings highlight the importance of study design when evaluating cancer risks and emphasize that robust statistical associations in retrospective studies do not necessarily imply causality.

## 1. Introduction

Gynecological cancers, which originate in a woman’s reproductive system, are among the most common cancers affecting females worldwide. According to GLOBOCAN 2022, cervical cancer is the fourth most common cancer among women globally, with an age-standardized incidence rate (ASR) of 14.1 per 100,000 [1]. It is followed by cancer of the endometrium (corpus uteri) (ASR 8.4) and of the ovary (ASR 6.7), making these three the most common gynecological cancers [1]. Cervical cancer is particularly widespread in low- and middle-income countries (LMICs), largely due to high risk of infection with Human Papillomavirus (HPV), and limited access to screening programs and HPV vaccination. These regions also experience the highest mortality rates for cervical cancer. In contrast, endometrial cancer is more common in high-income countries, especially in Europe [2]. Ovarian cancer incidence tends to be higher in high-income countries, whereas LMICs report reduced incidence [3,4]. Nevertheless, the highest ovarian cancer mortality rates are found in LMICs, often due to late-stage diagnosis, limited availability of specialized care, and inadequate treatment options [5,6].

In addition to implementing early detection strategies, promoting lifestyle modifications plays a vital role in reducing the global burden of gynecological cancers. HPV infection is the dominant cause of cervical cancer, and tobacco smoking plays an additional role. Various factors can influence the risk of developing endometrial and ovarian cancers, including obesity and related conditions such as diabetes, hypertension, and polycystic ovary syndrome (PCOS), as well as infections, reproductive history and treatments [7,8]. Several chemical agents have been suggested to increase the risk of female genital cancers. These include per- and polyfluoroalkyl substances (PFAS) [9], tetrachloroethylene, endocrine disruptors like bisphenol A (BPA), triclosan (commonly found in hygiene products), and methoxychlor (an insecticide), as well as heavy metals such as lead and cadmium [10,11,12]. Humans may be exposed to these harmful substances through occupational or environmental sources over the course of their lives. One of the agents to which women may be exposed is talc, which, due to its softness and absorbent properties, is used in personal care products such as baby powder, makeup, and other cosmetics. Its potential link to female genital cancers, particularly ovarian cancer, has been the subject of ongoing research. Earlier reviews and meta-analyses by Berge et al. in 2018 [13] and Taher et al. in 2019 [14] reported statistically significant associations between perineal talc use and ovarian cancer. However, findings varied depending on the histological subtype of the disease. A later review by Goodman et al. in 2020 [15], however, did not find sufficient evidence to support a causal link. Research on talc exposure and cervical cancer is very limited, whereas some studies have suggested a possible association between genital talc use and an increased risk of endometrial cancer, especially in postmenopausal women. Nonetheless, these findings have not been subject to a systematic review.

Therefore, we aimed to conduct a systematic review and meta-analysis to evaluate the association between talc exposure and the incidence and mortality of female genital cancers, specifically ovarian, cervical, and endometrial cancers. This study provides an updated synthesis of the literature through 2026, incorporates dose–response analyses (frequency and duration of exposure), and considers histologic subtypes of ovarian cancer to provide more detailed and clinically relevant insights than previous reviews.

## 2. Materials and Methods

### 2.1. Systematic Review

We conducted a search in MEDLINE (PubMed) and Scopus from inception to January 2026 to identify English, French, Italian, German, and Spanish-language cohort and case–control studies reported as peer-reviewed publications on the association between talc exposure and cancer risk (incidence and mortality). These two databases were selected because they provide broad and complementary coverage of biomedical and epidemiological literature and capture most relevant observational studies in this field; additionally, inclusion of other databases would not have substantially altered the search results. The review was reported following the Preferred Reporting Items for Systematic Reviews and Meta-Analyses (PRISMA) (Supplementary Table S1) statement. The protocol was registered in the International Prospective Register of Systematic Reviews (PROSPERO) (Registration number: CRD420261302988). The search strategy was designed using MeSH terms like: (“talc” OR “talcum” AND (“cancer” OR “malignant” OR “carcinoma” OR “tumor” OR “Leukemia” OR “Hematologic” OR “neoplasm” OR “myeloid”) (The complete search string is reported in Supplementary Table S2). This report is limited to studies reporting results of female genital cancers, including cervical, endometrial, and ovarian cancer. Results for other cancers are reported elsewhere [16].

**Figure 1:**
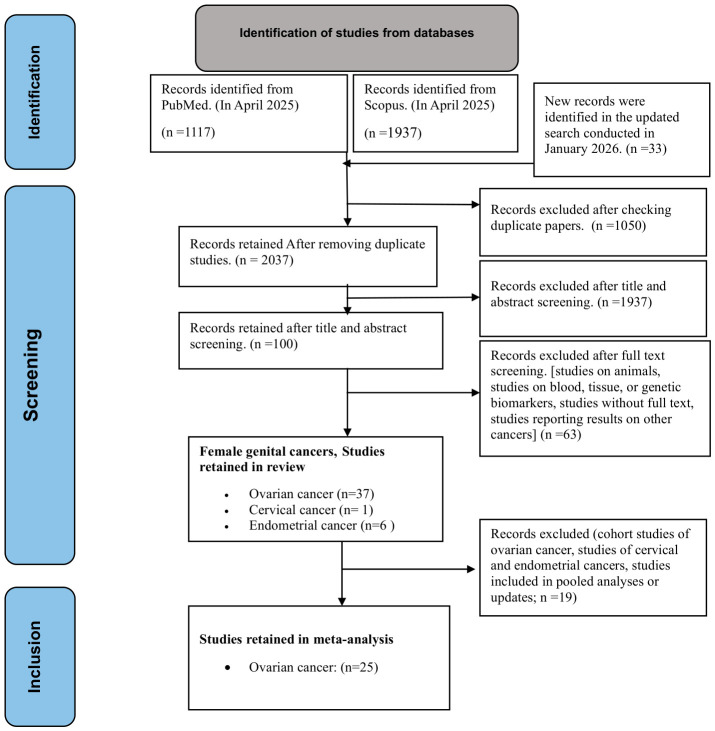
Selection of studies for inclusion in systematic review and meta-analysis.

Figure 1 shows the flow diagram of the literature search and study selection process.

Two reviewers (MSS and PB) independently reviewed the list of titles, abstracts, and full text of papers and their results were compared, and consensus was reached. The process included the following steps: (1) screening of the title and abstract of articles identified during the initial database search; (2) review of the full texts of articles included after step 1; (3) review of the references of articles included after step 2.

We included in the systematic review studies on humans that reported results on the association between exposure to talc not contaminated by asbestos or asbestiform fibers. These studies focused on individuals who were exposed to talc through cosmetics and hygiene products. Studies involving animals, blood, tissue, or genetic biomarkers, studies without full texts, and studies of workers who are mainly exposed to talc and other carcinogenic agents such as asbestos in occupational settings were excluded. In the case of multiple reports based on the same population, only the most informative report was included (generally the most recent one).

### 2.2. Data Extraction and Meta-Analysis

Relevant study characteristics were also extracted, such as author’s name, publication year, study design (case–control, and cohort), country, method of application for use of talc in consumer products (application to genital area, application to other areas, use on napkins, use on diaphragm, application after bathing), type of cancer (cervix, endometrium, and ovarian), behavior of ovarian tumor (borderline, and invasive), histology of ovarian cancer (serous, clear cell, endometroid, and mucinous), gender, frequency and duration of exposure, outcome (incidence, mortality), and the variables included in the analysis as potential confounders. (Supplementary Tables S3 and S4) Studies reporting odds ratio (OR), standardized mortality ratio (SMR), standardized incidence ratio (SIR), rate or risk ratio, hazard ratio (HR), and their 95% confidence intervals (CI), or sufficient data for their computation were included in the meta-analysis. Although these measures of association are not based on the same formulae, they all refer to the ratio of the disease in the exposed compared to the unexposed, and for practical purposes can be considered comparable and referred to as relative risk (RR). When articles provided the number of observed and expected cases or deaths but no SIRs or SMRs, these values and their respective 95% CIs were calculated. RRs were extracted for both ever-use and for specific levels, frequency, or duration of exposure. When studies included multiple frequency categories without ever vs. never, these were consolidated into a single ever-use group to ensure comparability.

### 2.3. Quality Assessment

We utilized a modified version of the Newcastle–Ottawa Scale (NOS) to assess the quality of papers included in our review [17]. Each case–control study was given a score ranging from 0 to 9, and each cohort study from 0 to 10. The mean (<7<) of the scores assigned independently was used to calculate the final NOS quality assessment score for this meta-analysis as high or low quality. In Supplementary Table S5, detailed quality assessment criteria for cohort and case–control studies are provided. In Supplementary Tables S3 and S4, the total quality score for each included study is reported.

### 2.4. Statistical Analysis

For cohort studies, because there was a limited number of individual studies available and all of them were included in large international pooling consortia, we extracted consortium-level pooled risk estimates rather than re-meta-analyzing individual cohort publications. These analyses used standardized covariate adjustment across cohorts, reducing methodological heterogeneity. Reconstructing pooled estimates from individual reports would have introduced inconsistencies in exposure classification and adjustment strategies.

For case–control studies, the main meta-analyses included results for any talc exposure versus no talc exposure and incidence or mortality based on a random-effects model [18]. This approach accounts for some heterogeneity between study-specific results, which may originate from differences in study designs or study populations. In studies where incidence and mortality were both reported, the primary meta-analysis comprised results on incidence, while each outcome was included in the relevant stratified analyses. Inter-study heterogeneity was measured by utilizing Cochran’s Q test and the I-square test [19]. The presence of publication bias and small study effects was assessed by visual inspection of the funnel plot and by applying the Egger test [20].

Stratified meta-analyses were conducted by countries (USA, other countries), years of publication (before 2004, after 2004), source/type of exposure (genital use, non-genital use, napkins, diaphragm, and after bathing), quality assessment score (low quality < 7, high quality > 7), histology and behavior of the tumor (serous, mucinous, endometroid, and clear cell).

To assess potential dose–response relationships between talc use and ovarian cancer risk, we conducted two-stage dose–response meta-analyses separately for duration and frequency of exposure. Only case–control studies were included, as cohort publications did not provide sufficient category-level data for trend estimation. Studies were eligible if they reported risk estimates for at least three exposure categories or provided sufficient data (relative risks with 95% confidence intervals and corresponding exposure levels, or distribution of cases and controls across categories) to estimate a quantitative trend. For each study, exposure levels were assigned using the midpoint of reported categories. For open-ended upper categories, the midpoint was estimated by assuming a width similar to the adjacent category, while never-use categories were assigned a value of zero. Frequency was standardized to applications per week and duration to years of use, when necessary. Then, first, we estimated study-specific log-linear trends using the generalized least-squares for trend (GLST) method, accounting for the correlation among risk estimates sharing a common reference group. Second, the resulting regression coefficients were pooled using a random-effects model (DerSimonian and Laird). Summary: relative risks were expressed per 10-year increase in duration of use and per 1 application per week increase in frequency. A log-linear relationship was assumed due to limited data for modeling non-linearity. Heterogeneity was assessed using Cochran’s Q and the I^2^ statistic [21].

A leave-one-out sensitivity analysis was performed to evaluate the influence of each individual case–control study related to ovarian cancer on the pooled effect estimate. The pooled effects were recalculated sequentially after omitting one study at a time, and results were presented as exponentiated ORs with 95% CIs.

All analyses were completed using Stata Statistical Software (STATA); Release 17 SE—Standard Edition (StataCorp LLC, College Station, TX, USA).

## 3. Results

### 3.1. Ovarian Cancer

#### 3.1.1. Cohort Studies

Since all available data from cohort studies (N = 4) [22,23,24,25] were included in the re-analysis by O’Brien et al. in 2020 [26], no meta-analysis of the results of these studies was performed. In that pooled analysis, the summary HR for ever-use of talc was 1.08 (95% CI: 0.99–1.17), with no intra-study heterogeneity; no trend was detected according to duration or frequency of use. The HR for long-term (≥20 years) compared to never use was 1.01 (95% CI, 0.82 to 1.25; *p* value = 0.57). Frequent use (1/week) versus none had an HR of 1.09 (95% CI, 0.97 to 1.23; *p* value for dose–response trend = 0.20).

#### 3.1.2. Case–Control Studies

A total of 25 (of the total 32) independent case–control studies [27,28,29,30,31,32,33,34,35,36,37,38,39,40,41,42,43,44,45,46,47,48,49,50,51,52,53,54,55,56,57,58] reporting data on the risk of ovarian cancer in talc-exposed populations were included in the meta-analysis. Seven studies were excluded from the meta-analysis due to database overlap. The analysis was first performed on all subtypes of ovarian cancer combined and all types of exposure source, then stratified according to the histology, behavior and source of exposure.

As shown in Supplementary Table S4, reporting the main characteristics of the studies included in our meta-analysis, most of them were performed in the USA (N = 21). All studies reported results on the incidence of ovarian cancer.

**Figure 2:**
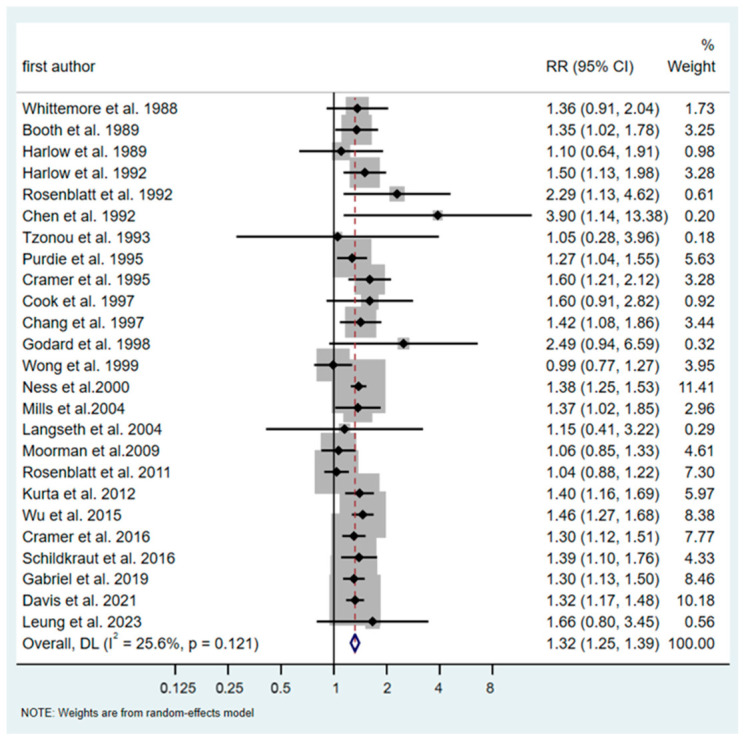
Forest plot (random-effects model) of results on the association between talc exposure and ovarian cancer (all types) in case–control studies [28,29,30,31,32,33,34,35,37,38,39,40,42,43,44,48,49,50,52,53,54,55,56,57,58].

**Figure 3:**
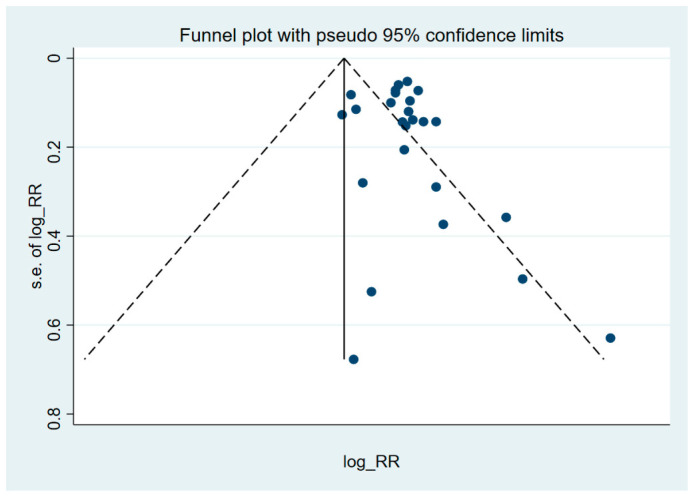
Funnel plot of results on the association between talc exposure and ovarian cancer (all types) in case–control studies. Solid circles represent individual studies, the solid vertical line represents the pooled overall effect estimate, and the dashed diagonal lines indicate the pseudo 95% confidence limits. (Egger test, *p*-value = 0.29).

The overall RR for all types of ovarian cancer and ever talc exposure was 1.32 (95% CI: 1.25–1.39), with limited evidence of heterogeneity (*p* = 0.12) (Figure 2). No evidence of publication bias was identified according to the funnel plot (*p*-value of Egger test, 0.29) (Figure 3).

Table 1 displays the findings of stratified meta-analyses based on selected characteristics. There was no heterogeneity in the summary results after stratification by geographic region (USA vs. other countries, *p* = 0.60) and by year of publication (before 2004 vs. 2004 or later, *p* = 0.38).

There was heterogeneity of results according to the circumstance of talc exposure (*p* = 0.01), with supportive results for the use of talc directly on the genital area (RR 1.38, 95% CI: 1.31–1.44) and using talc only after bathing (RR 1.30, 95% CI: 1.08–1.56) compared to other circumstances of exposure, such as through napkins or diaphragm.

The analyses based on tumor behavior showed comparable results for invasive vs. borderline tumors (p for heterogeneity = 0.57), whereas those by histology suggested a stronger association with serous (RR 1.36, 95% CI: 1.26–1.47) and endometroid type (RR 1.35, 95% CI: 1.13–1.61) compared to other types (p for heterogeneity = 0.05).

Nine case–control studies reported results on the risk of ovarian cancer by duration of talc exposure (Supplementary Table S6). Table 2 shows the results of the two-stage dose–response meta-analysis. The summary RR for a 10-year increase in the duration of talc exposure was 1.09 (95% CI: 1.06–1.12). The RR of individual studies ranged from 0.99 to 1.77 (p of heterogeneity 0.60).

Four case–control studies reported results on the risk of ovarian cancer by frequency of talc exposure (Supplementary Table S7). Categories of use were re-classified in terms of the number of applications per week. Table 3 shows the results of the two-stage dose–response meta-analysis: The summary RR for an increase of one time/week use of talc was 1.04 (95% CI: 1.01–1.07; p of heterogeneity = 0.23).

The leave-one-out sensitivity analysis for case–control studies related to ovarian cancer demonstrated that the pooled effect estimates remained highly consistent regardless of which study was excluded. The recalculated odds ratios ranged from approximately 1.31 to 1.34, with all 95% confidence intervals remaining statistically significant (*p* < 0.001). No single study had a disproportionate influence on the overall estimate, indicating that the meta-analysis results are robust and not driven by any individual study (Supplementary Figure S1).

#### 3.1.3. Overall Pooled Estimate (Cohort + Case–Control)

When the single cohort study was combined with case–control studies, the overall pooled relative risk (RR) was 1.24 (95% CI: 1.19–1.30). This provides a summary measure across available study designs, although the majority of data comes from case–control studies.

### 3.2. Endometrial Cancer

#### 3.2.1. Cohort Studies

We identified five cohort studies that reported results related to endometrial cancer; however, one of them, by O’Brien et al. in 2021 [59], summarized results of three studies [60,61,62] in a pooled analysis. They found an HR of 1.01 (95% CI: 0.94–1.09) for the ever-use of talc. In 2024, O’Brien et al. [63] published another report with updated data from the sister cohort study, whose demonstrated results were consistent with the previous pooled analysis (Supplementary Table S3).

#### 3.2.2. Case–Control Studies

In terms of case–control studies, the only relevant research is by Neill et al. in 2012 [64], which examined an Australian population. This study found no association between talc use and the risk of endometrial cancer, reporting an OR of 0.88 (95% CI: 0.68–1.14). Given the limited number of studies available, a meta-analysis of these results was not conducted (Supplementary Table S4).

### 3.3. Cervical Cancer

Only one cohort [65] study was identified that evaluated cervical cancer risk in relation to adolescent talc use. The results showed an overall RR of 0.95 (95% CI 0.76–1.19) for cervical cancer associated with talc use. Additionally, no associations were found between talc use or frequency during the ages of 10 to 13 (Supplementary Table S3).

## 4. Discussion

According to our results, the available cohort studies do not show an association between talc use and the risk of ovarian cancer, whereas our meta-analysis of case–control studies indicates a positive association. In cohort studies, the prevalence of ever-use among ovarian cancer cases was around 44%, whereas in many case–control studies it ranged from 52% to 59% difference may partly reflect recall bias, since case–control studies often rely on participants’ retrospective self-reporting of past exposure, which could inflate observed associations. Case–control designs also have strengths, such as the ability to study rare outcomes and collect detailed exposure histories, whereas cohort studies capture exposure prospectively, reducing recall bias but sometimes limiting statistical power for uncommon outcomes or detailed exposure patterns. Together, these differences highlight that estimates of the possible risk should be interpreted cautiously, considering the methodological strengths and limitations of each study design. In several cohorts, talc exposure was assessed only at baseline, potentially leading to nondifferential misclassification if exposure patterns changed over time. Additionally, prospective questionnaires may not have captured detailed information on application methods, duration, or frequency with the same granularity as case–control interviews. Ovarian cancer is a relatively rare outcome, and even large cohorts may have limited power for analyses by histologic subtype or long-term exposure categories. Cohort studies were already combined previously, and only one could be combined with case–control studies; in that case, the pooled RR was 1.24, 95% CI: 1.19–1.30. Regarding endometrial and cervical cancer, no association was observed; however, the limited number of available studies precludes firm conclusions.

While the exact mechanism by which talc may contribute to cancer is not fully understood, in vivo and in vitro studies suggest several biological processes that could be involved. For instance, (1) talc exposure may trigger inflammation, a well-known factor in cancer development, and induce oxidative stress, which can lead to DNA damage that promotes the onset of cancer [46,66]. (2) Some studies have detected talc particles in ovarian tumors, supporting the hypothesis that talc could play a role in tumor development [67]. (3) Talc may influence cellular behavior by promoting cell proliferation and other changes that could contribute to cancer. The proposed carcinogenic pathway suggests that talc particles can travel through the reproductive tract, passing through the vagina, cervix, and uterus, to reach the fallopian tubes and ovaries. However, this hypothesis requires further investigation [68]. It would be important to study other components of body powders, such as corn starch, which may also provoke inflammatory responses [69,70]. Despite limitations in current experimental models, exploring the effects of these substances on inflammation in different parts of the genital tract, such as the cervix or endometrium, could help identify key biological markers and intermediate processes relevant to cancer risk. Furthermore, attention should also be given to additional agents present alongside talc in cosmetic products.

Published reports on talc exposure and its association with female genital cancers show varying results, with a primary focus on ovarian cancer and limited evidence for other types. For instance, in 2017, Penninkilampi et al. [71] conducted a meta-analysis that included 24 case–control studies and 3 cohort studies examining the relationship between perineal talc use and ovarian cancer. The analysis indicated that any use of talc in the perineal area was linked to an increased risk of ovarian cancer. However, this association was not found when the analysis was limited to cohort studies alone [71]. Then, in 2018, Bergen et al. conducted a meta-analysis that included the same number of studies as a previous analysis and supported its findings. They also identified a weak trend indicating that the relative risk of ovarian cancer increases with the duration and frequency of genital talc use [13]. In 2019, Taher et al. published a critical review and meta-analysis exploring the link between perineal talc use and ovarian cancer. Their key findings included the following: first, perineal talc application might be a potential cause of ovarian cancer in humans; second, women who are Hispanic or White, as well as both premenopausal and postmenopausal women receiving hormone therapy, are at higher risk; and finally, the use of oral contraceptives, tubal ligation, hysterectomy, and breastfeeding may provide protective effects [14]. Two systematic reviews and meta-analyses published in 2021 and 2022 confirmed a weak association between the use of genital talc and the risk of ovarian cancer, particularly with frequent application to the perineal area. However, the composition of body powders can vary significantly, which makes it difficult to quantify exposure [72,73].

In contrast, a study by Goodman et al. in 2020 concluded that the existing evidence does not support a causal relationship between perineal talc use and ovarian cancer [15]. In 2023, Lynch et al. conducted a systematic review examining the relationship between talc exposure and cancers of the female reproductive tract, including ovarian, cervical, and endometrial cancers. They did not perform a meta-analysis but concluded that there is suggestive evidence indicating no association between perineal talc application and ovarian cancer at exposure levels relevant to humans. Additionally, they found no association with endometrial cancer and deemed the evidence regarding cervical cancer as insufficient due to the small and mostly inconclusive body of literature [74]. Then, in 2024, Boon et al. conducted another systematic review focusing on the epidemiological evidence concerning various sources of talc exposure and cancer risk. They noted that consistent associations with ovarian cancer were only observed in case–control studies, which may be affected by recall and other biases. No associations were identified for cervical or endometrial cancers. The review ultimately concluded that epidemiological studies do not support a causal link between occupational, medicinal, or personal talc exposure and any form of cancer in humans [75]. Finally, a recent comprehensive review by Tsokkou and co-authors concluded that the epidemiological evidence remains inconclusive, noting that positive associations are largely confined to retrospective studies and that causality has not been established [76].

According to our sub-analysis of case–control studies, individuals who regularly use talc directly in the genital area, or after bathing, may be at higher risk compared to those who use talc in products like napkins, diaphragms, or apply it to non-genital areas. Also, over the years, the results have remained consistent, with similar results observed before and after 2004. Although most studies were conducted in the USA, all regions investigated indicated a risk associated with talc use. When we examine the histology of ovarian cancer, we find significant differences between the serous and endometrioid types compared to others. However, there is no distinction between borderline and invasive tumor behavior. Previous research has highlighted that the histological type of ovarian cancer may be a significant factor. For instance, Cramer et al. in 2016 reported that certain histologic subtypes of epithelial ovarian cancer, including serous and mucinous borderline tumors, as well as invasive serous and endometrioid tumors, are more likely to be associated with talc use [53]. Additionally, Berge et al. in 2018 showed that serous carcinoma was the only histologic type showing a detectable association with talc use (RR: 1.24; 95% CI: 1.15–1.34) [13].

Our study, which extends these previous reviews, has some strengths. First, we were able to analyze the results from different talc products while also considering the duration and frequency of use. Additionally, we focused on the clinical characteristics of tumors, such as histology and behavior, particularly in relation to ovarian cancer. Another important aspect is that our meta-analysis utilized case–control studies, which typically include a diverse range of potentially relevant confounders in their models. However, it is worth noting that many studies did not adjust for these factors. Furthermore, both cohort and case–control studies have potential limitations, but a key distinction is that case–control studies assess exposure retrospectively, which may increase the risk of recall bias, whereas cohort studies collect exposure prospectively, minimizing this particular source of bias, although they did not provide information on critical details of use. For example, a recent analysis of the Sister Study demonstrated that awareness of disease status may influence exposure reporting, potentially inflating risk estimates in case–control studies [77]. Although quantitative bias analysis could theoretically be applied, the absence of validation data on the sensitivity and specificity of self-reported talc exposure precluded such adjustment. Finally, although statistical heterogeneity was low in our meta-analysis of case–control studies (I^2^ = 25.6%), this does not imply conceptual homogeneity. Studies varied in exposure definitions, assessment methods, populations, and time periods. Genital talc use was generally self-reported, but frequency, duration, and timing differed, with most relying on retrospective recall. Geographic and birth-cohort differences may reflect changes in product composition and use patterns. Adjustment for reproductive, hormonal, and lifestyle factors also varied. These differences are not fully captured by statistical heterogeneity and complicated interpretation of pooled estimates, particularly across study designs. The leave-one-out sensitivity analyses showed that the pooled estimate from case–control studies remained stable when individual studies were excluded, with odds ratios varying only minimally and all remaining statistically significant. This demonstrates that the observed positive association is consistent across the body of case–control evidence and not driven by any single study. However, while the statistical robustness of these findings is clear, the retrospective nature of case–control studies means they are susceptible to recall and selection biases. Therefore, despite the consistency of the association, these methodological limitations prevent us from interpreting the results as conclusive evidence of causality.

Nonetheless, one of the main limitations of our study is the low number of cohort studies specifically examining ovarian cancer, as well as cervical and endometrial cancers. This absence prevented us from conducting a meta-analysis and highlights the need for future research in this area. Additionally, many studies did not account for co-exposure to other agents used in the production of products like napkins. In our analysis, we focused solely on studies involving talc used in cosmetic products, particularly for the genital area. Finally, major studies came from the USA compared to other regions, although the results were similar, particularly related to ovarian cancer. It may be beneficial to conduct further research on occupational exposure or other sources of talc exposure.

## 5. Conclusions

In conclusion, available cohort studies do not support an association between ever-use of talc and ovarian cancer risk. Several case–control studies, as well as our meta-analysis of these studies, reported a positive association between talc use and ovarian cancer. However, case–control studies are more susceptible to recall bias and selection bias, whereas cohort studies collect exposure information prospectively and follow participants over time, reducing these biases. For this reason, cohort study findings may provide a more reliable estimate of the true association. Results for endometrial and cervical cancer did not suggest any association with talc exposure.

The explanation of epidemiological evidence on talc exposure has important implications for public health and regulatory decision-making. Recent concerns have been raised regarding the recent IARC classification of talc carcinogenicity, where pure talc was reclassified from Group 2B as Possibly carcinogenic to humans to Group 2 A as Probably carcinogenic to humans [78,79], particularly with respect to the relative weighting of different study designs. Korchevskiy and Wylie [70] argued that this classification may place disproportionate emphasis on hazard identification based largely on case–control studies, while underweighting null findings from cohort studies and evidence from quantitative risk assessments. Further research, particularly well-designed cohort studies, is needed to clarify the potential risks, especially for cervical and endometrial cancers, where evidence remains scarce.

## Figures and Tables

**Table 1:** Results of the meta-analysis of case–control studies on ever-use of talc and ovarian cancer risk stratified by country, year of publication, exposure source, histology type, and tumor behavior.

Characteristic	N Risk Estimates	RR, 95% CI	*p* Heterogeneity
Country			
USA	17	1.31 (1.23–1.39)	0.60
Other countries	8	1.36 (1.19–1.55)	
Year of publication			
<2004	14	1.37 (1.25–1.49)	0.34
≥2004	11	1.29 (1.20–1.39)	
Quality score			
Low quality < 7	12	1.33(1.24–1.44)	0.47
High quality ≥ 7	13	1.28(1.18–1.39)	
Exposure source			
Genital use	15	1.38 (1.31–1.44)	0.01
Nongenital use	3	1.21 (0.94–1.54)	
Napkins	7	1.21 (0.84–1.74)	
Diaphragm	4	0.74 (0.52–1.05)	
After bathing	4	1.30 (1.08–1.56)	
Histology type			
Serous	9	1.36 (1.26–1.47)	0.05
Mucinous	5	1.01 (0.80–1.27)	
Endometrioid	4	1.35 (1.13–1.61)	
Clear cell	2	1.04 (0.75–1.45)	
Tumor behavior			
Borderline	8	1.25 (1.08–1.46)	0.57
Invasive	10	1.18 (1.02–1.36)	

RR, relative risk; CI, confidence interval.

**Table 2:** Dose–response meta-analysis of case–control studies on duration of talc use and risk of ovarian cancer in case–control studies.

Reference	RR	95% CI	Weight %
Chang et al., 1997 [38]	1.03	0.94–1.12	9.36
Wong et al., 1999 [40]	0.99	0.86–1.14	3.66
Mills et al., 2004 [43]	1.09	0.96–1.23	4.53
Rosenblatt et al., 2011 [49]	1.04	0.94–1.16	6.06
Cramer et al., 2016 [53]	1.09	1.03–1.14	27.28
Schildkraut et al., 2016 (Non-genital use) [54]	1.10	0.96–1.26	3.73
Schildkraut et al., 2016 (Genital use) [54]	1.15	1.04–1.27	6.33
Gabriel et al., 2019 [55]	1.13	1.05–1.21	14.11
Davis et al., 2021 [56]	1.10	1.04–1.17	22.44
Leung et al., 2023 [58]	1.77	0.62–5.02	0.06
Overall	1.09	1.06–1.12	100

RR, relative risk for a 10-year increase in duration of exposure; CI, confidence interval.

**Table 3:** Dose–response meta-analysis of case–control studies on frequency of talc use and risk of ovarian cancer in case–control studies.

Reference	RR	95% CI	Weight %
Whittemore et al., 1988 [28]	1.06	0.99–1.12	14.55
Chang et al., 1997 [38]	1.00	0.95–1.05	17.63
Mills et al., 2004 [43]	1.10	1.02–1.18	11.07
Cramer et al., 2016 [53]	1.04	1.02–1.06	39.11
Overall	1.04	1.01–1.07	100

RR, relative risk for a 1-time/week increase in frequency of exposure: CI, confidence interval.

## Data Availability

No new data were created or analyzed in this study. Data sharing is not applicable to this article.

## Supplementary Materials

The following supporting information can be downloaded at: https://www.mdpi.com/article/10.3390/cancers18101589/s1, Table S1: PRISMA Checklist and PRISMA Abstract Checklist; Table S2: Detailed search strategy used on the different databases; Table S3: Selected characteristics of cohort studies on talc exposure and ovarian, cervical and endometrial cancer; Table S4: Selected characteristics of case–control studies on talc exposure and ovarian, cervical** and endometrial cancer; Table S5: Modified version of the Newcastle–Ottawa Scale (NOS) for case–control and cohort studies adopted for quality assessment; Table S6: Relative risk of ovarian cancer by duration of exposure to talc in case–control studies; Table S7: Relative risk of ovarian cancer by frequency of exposure to talc in case–control studies; Figure S1: Leave-one-out sensitivity analysis of the association between case–control studies related to talc use and ovarian cancer risk.

## Abbreviations

The following abbreviations are used in this manuscript:

ASR	Age-standardized incidence rate
BPA	Bisphenol A
CI	Confidence intervals
HDI	Human Development Index
HR	Hazard ratio
IARC	International Agency for Research on Cancer
LMICs	Low- and middle-income countries
NOS	Newcastle–Ottawa Scale
OR	Odds ratio
PFAS	Per- and polyfluoroalkyl substances
PCOS	Polycystic ovary syndrome
RR	Risk ratio, rate ratio
SMR	Standardized mortality ratio
SIR	Standardized incidence ratio
