# Supplementary material for: Talc Exposure and Risk of Ovarian Cancer: A Systematic Review and Meta-Analysis with Limited Evidence on Cervical and Endometrial Cancers
Source: Cancers (Basel). 2026 May 13;18(10):1589. doi: 10.3390/cancers18101589 (PMC13204818; doi:10.3390/cancers18101589)
Supplement: Supplementary file 1 [file cancers-18-01589-s001.zip › cancers-4257711-supplementary.pdf]

Supplementary Materials

Table S1. PRISMA Checklist and PRISMA Abstract Checklist.

Table S2. Detailed search strategy used on the different databases.

Table S3. Selected characteristics of cohort studies on talc exposure and ovarian, cervical and endometrial cancer.

Table S4. Selected characteristics of case-control studies on talc exposure and ovarian, cervical** and endometrial cancer.

Table S5. Modified version of the Newcastle-Ottawa Scale (NOS) for case-control and cohort studies adopted for quality assessment.

Table S6. Relative risk of ovarian cancer by duration of exposure to talc in case-control studies.

Table S7. Relative risk of ovarian cancer by frequency of exposure to talc in case-control studies.
Figure S1: Leave-one-out sensitivity analysis of the association between case control studies related to talc use and ovarian cancer risk.

Table S1. PRISMA Checklist and PRISMA Abstract Checklist.

Section and Topic	Item #	Checklist item	Location where item is reported
**TITLE**			
Title	1	Identify the report as a systematic review.	P1
**ABSTRACT**			
Abstract	2	See the PRISMA 2020 for Abstracts checklist.	P2
**INTRODUCTION**			
Rationale	3	Describe the rationale for the review in the context of existing knowledge.	P4
Objectives	4	Provide an explicit statement of the objective(s) or question(s) the review addresses.	P4
**METHODS**			
Eligibility criteria	5	Specify the inclusion and exclusion criteria for the review and how studies were grouped for the syntheses.	P5
Information sources	6	Specify all databases, registers, websites, organisations, reference lists and other sources searched or consulted to identify studies. Specify the date when each source was last searched or consulted.	P5
Search strategy	7	Present the full search strategies for all databases, registers and websites, including any filters and limits used.	P5, Supplementary Table 1
Selection process	8	Specify the methods used to decide whether a study met the inclusion criteria of the review, including how many reviewers screened each record and each report retrieved, whether they worked independently, and if applicable, details of automation tools used in the process.	P5
Data collection process	9	Specify the methods used to collect data from reports, including how many reviewers collected data from each report, whether they worked independently, any processes for obtaining or confirming data from study investigators, and if applicable, details of automation tools used in the process.	P5
Data items	10a	List and define all outcomes for which data were sought. Specify whether all results that were compatible with each outcome domain in each study were sought (e.g. for all measures, time points, analyses), and if not, the methods used to decide which results to collect.	P5

Section and Topic	Item #	Checklist item	Location where item is reported
	10b	List and define all other variables for which data were sought (e.g. participant and intervention characteristics, funding sources). Describe any assumptions made about any missing or unclear information.	P5
Study risk of bias assessment	11	Specify the methods used to assess risk of bias in the included studies, including details of the tool(s) used, how many reviewers assessed each study and whether they worked independently, and if applicable, details of automation tools used in the process.	P5,6
Effect measures	12	Specify for each outcome the effect measure(s) (e.g. risk ratio, mean difference) used in the synthesis or presentation of results.	P6
Synthesis methods	13a	Describe the processes used to decide which studies were eligible for each synthesis (e.g. tabulating the study intervention characteristics and comparing against the planned groups for each synthesis (item #5)).	P6
	13b	Describe any methods required to prepare the data for presentation or synthesis, such as handling of missing summary statistics, or data conversions.	P6
	13c	Describe any methods used to tabulate or visually display results of individual studies and syntheses.	P6
	13d	Describe any methods used to synthesize results and provide a rationale for the choice(s). If meta-analysis was performed, describe the model(s), method(s) to identify the presence and extent of statistical heterogeneity, and software package(s) used.	P6
	13e	Describe any methods used to explore possible causes of heterogeneity among study results (e.g. subgroup analysis, meta-regression).	P6
	13f	Describe any sensitivity analyses conducted to assess robustness of the synthesized results.	P6
Reporting bias assessment	14	Describe any methods used to assess risk of bias due to missing results in a synthesis (arising from reporting biases).	P6
Certainty assessment	15	Describe any methods used to assess certainty (or confidence) in the body of evidence for an outcome.	P6
**RESULTS**			
Study selection	16a	Describe the results of the search and selection process, from the number of records identified in the search to the number of studies included in the review, ideally using a flow diagram.	P7, supplementary figure 1
	16b	Cite studies that might appear to meet the inclusion criteria, but which were excluded, and explain why they were excluded.	supplementary figure 1
Study characteristics	17	Cite each included study and present its characteristics.	P7
Risk of bias in studies	18	Present assessments of risk of bias for each included study.	P7
Results of individual studies	19	For all outcomes, present, for each study: (a) summary statistics for each group (where appropriate) and (b) an effect estimates and its precision (e.g. confidence/credible interval), ideally using structured tables or plots.	P7, Supplementary Table 3
Results of syntheses	20a	For each synthesis, briefly summarise the characteristics and risk of bias among contributing studies.	P67, Supplementary Table 3
	20b	Present results of all statistical syntheses conducted. If meta-analysis was done, present for each the summary estimate and its precision (e.g. confidence/credible interval) and measures of statistical heterogeneity. If comparing groups, describe the direction of the effect.	P7, Figures 1, 2, and 3
	20c	Present results of all investigations of possible causes of heterogeneity among study results.	P7, Figure 4
	20d	Present results of all sensitivity analyses conducted to assess the robustness of the synthesized results.	P7, 8, Supplementary table 4
Reporting biases	21	Present assessments of risk of bias due to missing results (arising from reporting biases) for each synthesis assessed.	P7
Certainty of evidence	22	Present assessments of certainty (or confidence) in the body of evidence for each outcome assessed.	P7,8

Section and Topic	Item #	Checklist item	Location where item is reported
**DISCUSSION**			
Discussion	23a	Provide a general interpretation of the results in the context of other evidence.	P9
	23b	Discuss any limitations of the evidence included in the review.	P10
	23c	Discuss any limitations of the review processes used.	P10
	23d	Discuss implications of the results for practice, policy, and future research.	P10
**OTHER INFORMATION**			
Registration and protocol	24a	Provide registration information for the review, including register name and registration number, or state that the review was not registered.	P1, 5
	24b	Indicate where the review protocol can be accessed, or state that a protocol was not prepared.	NA
	24c	Describe and explain any amendments to information provided at registration or in the protocol.	NA
Support	25	Describe sources of financial or non-financial support for the review, and the role of the funders or sponsors in the review.	P11
Competing interests	26	Declare any competing interests of review authors.	P11
Availability of data, code and other materials	27	Report which of the following are publicly available and where they can be found: template data collection forms; data extracted from included studies; data used for all analyses; analytic code; any other materials used in the review.	P11

Section and Topic	Item #	Checklist item in abstract	Reported (Yes/No)
**TITLE**			
Title	1	Identify the report as a systematic review.	Yes
**BACKGROUND**			
Objectives	2	Provide an explicit statement of the main objective(s) or question(s) the review addresses.	Yes
**METHODS**			
Eligibility criteria	3	Specify the inclusion and exclusion criteria for the review.	Yes
Information sources	4	Specify the information sources (e.g. databases, registers) used to identify studies and the date when each was last searched.	Yes
Risk of bias	5	Specify the methods used to assess risk of bias in the included studies.	Yes
Synthesis of results	6	Specify the methods used to present and synthesise results.	Yes
**RESULTS**			
Included studies	7	Give the total number of included studies and participants and summarise relevant characteristics of studies.	Yes
Synthesis of results	8	Present results for main outcomes, preferably indicating the number of included studies and participants for each. If meta-analysis was done, report the summary estimate and confidence/credible interval. If comparing groups, indicate the direction of the effect (i.e. which group is favoured).	Yes

Table S2. Detailed search strategy used on the different databases.

Database	Search string
PubMed	Search: ("talc"[Text Word] OR "talcum"[Text Word]) AND ("cancer"[Text Word] OR "malignant"[Text Word] OR "carcinoma"[Text Word] OR "tumor"[Text Word] OR "Leukemia"[Text Word] OR "Hematologic"[Text Word] OR "neoplasm"[Text Word] OR "myeloid"[Text Word])
Scopus	(TITLE-ABS-KEY ("talc") OR TITLE-ABS-KEY ("talcum")) AND (TITLE-ABS-KEY("cancer") OR TITLE-ABS-KEY("malignant") OR TITLE-ABS-KEY("carcinoma") OR TITLE-ABS-KEY("neoplasm") OR TITLE-ABS-KEY("tumor") OR TITLE-ABS-KEY("myeloid") OR TITLE-ABS-KEY("lymphoma") OR TITLE-ABS-KEY("Hematologic"))

Table S3. Selected characteristics of cohort studies on talc exposure and ovarian, cervical and endometrial cancer.

Reference	Country	Exposure type (measurement method)	Source population	Period of enrolment	N subjects/N ovarian cancer cases	Factors adjusted for	Quality score
			**Ovarian cancer**				
Gertig et al.2000 [22]	USA	Perineal talc use, Napkins, overall (self-reported interview)	Nurses' Health Study	1976	78,630/307	A, P, OC, BMI, TL, Smk, HRT	9
Gates et al.2010 [23]	USA	Talc use (self-reported interview)	Nurses' Health Study and Nurses' Health Study-II	1976/1989	238130/924	OC, BMI, TC, Smk, HRT, A, Men, BF	9
Houghton et al.2014 [24]	USA	Combined ever powder use (genital, napkins diaphragms), Genital powder, Napkins, Diaphragm (self-reported interview)	Women's Health Initiative Observational Study (WHI-OS)	1993-1998	61576 /429	A, R, OC, HRT, FH, BMI, Smk, TL, P	7.5
Gonzalez et al.2016 [25]	USA	Genital talc (self-reported interview)	The Sister Study	2003	9023/167	R, BMI, P, OC, Men	6.5
O'Brien et al.2020 [26]	USA	Genital talc (self-reported interview)	Pooled analysis of Nurses' Health Study, Nurses' Health; Study II, Sister Study, Women's Health Initiative Observational	1976/1989/2003-2009/1993-1998	252,745/2168	R, Ed, BMI, P, OC, TL, Hys, Men, HRT	9
			**Cervical cancer**				
O Brien et al, 2021 [59]	USA	Genital talc (self-reported interview)	The Sister Study	2003	49,302/523	A, R, SES, Smk	8
			**Endometrial cancer**				
Karageorgi et al, 2010 [60]	USA	Genital talc (self-reported interview)	Nurses' Health Study	1976	66,088/599	A, OC, BMI, SMK, diabetes, FH, Men	9
Crawford et al, 2012 [61]	USA	Genital talc (self-reported interview)	Women's Health Initiative Observational Study (WHI-OS)	1993-1998	61576/435	A, R, BMI, Smk, LB, OC, HRT	7
O'Brien et al, 2019 [62]	USA	Genital talc (self-reported interview)	The Sister Study	2003	33,858/274	R, Ed, BMI, Men, P, OC, Smk, HRT, Alc	8
O'Brien et al, 2021 [65]	USA	Genital talc (self-reported interview)	Pooled analysis of Nurses' Health Study, Nurses' Health; Study II, Sister Study, Women's Health Initiative Observational	1976/1989/2003-2009/1993-1998	2 937 309/3162	BMI, R, Ed, P, Smk, OC, Men, HRT, BO	9
O'Brien et al, 2024 [63]	USA	Genital talc (self-reported interview)	The sister study	2003	50,800/433	R, Ed, BMI, A, Men, SMK, HRT, P, Alc, GR	8

A, age; P, parity; OC, oral contraceptive; BMI, body mass index; TL, tubal ligation; Smk, tobacco smoking; HRT, hormone replacement therapy; Men, mesopausal status; BF, breast feeding; R, race, Ed, education, FH, family history of ovarian cancer; Hys, hysterectomy; Alc, Alcohol; GR, geographic region ; BO, bilateral oophorectomy; LB, number of live births; SES, socioeconomic status. We excluded two studies from our project because they did not focus on talc exposure

exactly and they only reported by job title and possibility exposure to talk.1: Langseth et al, 1999 working on occupational (pulp and paper industry), 2: Bulbulyan et al, 1999 working on occupational (All printing plantempl).

Table S4. Selected characteristics of case-control studies on talc exposure and ovarian, cervical** and endometrial cancer.

Reference	Country	Exposure type (measurement method)	Period of enrolment	Source of controls	# cases/ controls	Factors adjusted for‡	Quality score
			Ovarian cancer				
* Cramer et al. 1982 [27]	USA	Any perineal exposure (self-report in-person interview)	1978-1981	Population-based controls	215/215	P, Men	7
Whittemore et al.1988 [28]	USA	Perineum only, pads only, diaphragm only, any exposure (self-report, Structured in-home interviews)	1983-1985	Hospital patients without cancer, and general population	188/259	P, OC	6
Booth et al.1989 [29]	UK	Genital use (self report, standard interviewer-administered questionnaire)	1978-1983	Hospital-based controls	235/451	SES	6.5
Harlow et al.1989 [30]	USA	Any genital exposure, Diaphragm only, After bathing only, Napkins only(self-report, in-person interviews)	1980 -1985	Population-based controls	116/158	P, OC	6.5
Harlow et al.1992 [31]	USA	Any genital exposure	1984-1987	Population-based controls	235/239		6
Rosenblatt et al.1992 [32]	USA	Any genital exposure, Genital bath talc, Napkins (self-report, structured questionnaire administered by interview)	1981-1985	Hospital-based controls	77/46		6
Chen et al.1992 [33]	China	Talc-containing dusting powder to the lower abdomen and perineum (self-reported information collected in face-to-face interviews.)	1984-1986	Community-based controls	112/224	Ed, P	7
Tzonou et al.1993 [34]	Greece	Talc in perineum (self-reported interview in hospital)	1989-1991	Hospital-based controls	189/200	Ed, BMI, Men, P, Smk, Cof, Alc, HD, Med	7.5
† Purdie et al. 1995 [35]	Australia	Talc use around abdomen and perineum (self-reported information collected in face-to-face interviews.)	1990-1993	Population-based controls	824/860		6
Cramer et al.1995 [36]	USA	Talc use (self-reported information collected in face-to-face interviews.)	1984-1987/ 1978-1981	Population-based controls	450/454		5
Cook et al.1997 [37]	USA	Any perineal talcum use (self-reported information collected in face-to-face interviews.)	1986-1988	Population-based controls	313/422	OP	6.5
Chang et al.1997	Canada	Any talc exposure,	1989-1992	Population-based	450/564	OC, P, BF, TL,	8

[38]		Napkins, After bathing only(self-reported information collected in face-to-face interviews.)		controls		Hy FH	
Godard et al.1998 [39]	Canada	Perineum (self-reported interview)	1995-1996	Population-based controls	170/170		6.5
Wong et al.1999 [40]	USA	Any talc exposure Napkins Genital or thigh area (Self-administered questionnaire)	1982-1995	Population-based controls	499/755	P, OC, Smk, FH, AM, Men, In, Ed, Reg, TL, Hys	7.5
* Cramer et al.1999 [41]	USA	Genital use talc (self-report in person interview)	1992-1997	Population-based controls	563/523	TL, OP	7
Ness et al.2000 [42]	USA	Any talc exposure, Feet, body, Genital/rectal, Napkins, Underwear, Diaphragm, Male partner (self-report interview)	1994-1998	Population-based controls	767/1367	P, R, FH, OC, TL, Hy, BF	7
Mills et al.2004 [43]	USA	Perineal talc use	2000-2001	Population-based controls	256/1122	R, OC, BF	7
Langseth et al.2004 [44]	Norway	Occupational (pulp and paper industry) (this result wasn't included in analysis) Talc by personal use (diapers, sanitary napkins, no genital area or husband's use in genital area.) (this result included in analysis) (self-report interview)	1953-1999	Population-based controls	46/184		6
* Cramer et al.2005 [45]	USA	Any talc exposure Body use Genital use (self-report interview)	1998-2003	Population-based controls	705/668	P, R, Rel	6.5
* Merritt et al.2008 [46]	Australia	Perineal talc use (self-report interview)	2002-2005	Population-based controls	1576/1509	Ed, P, OC	6.5
* Gates et al.2008 [47]	USA	Genital use (self-report interview)	1992-1997/1976-2004/1976	Population-based controls	1175/1202		7
Moorman et al.2009 [48]	USA	Talc use (self-report in person interview)	1999–2008	Population-based controls	1086/1057		6
Rosenblatt et al.2011 [49]	USA	Any talc exposure, Powder after bathing, Napkins, Diaphragm, Deodorant sprays(self-report in person interview)	2002-2005	Population-based controls	812/1313	Reg, P, OC	8
Kurta et al.2012 [50]	USA	Perineal talc use(self-report in person interview)	2003-2008	Population-based controls	902/1802	R, Ed	6.5
* Terry et al.2013 [51]	USA	Any talc exposure, No genital use, Genital use (self-report in person interview)	2010	Population-based controls	8525/9859	OC, P, TL, BMI, R	7.5
Wu et al.2015 [52]	USA	Genital use (self-report in person interview)	2003-2008	Population-based controls	1701/2391	R, Men AM, HRT, BMI, In, Ed	8
Cramer et al.2016	USA	Powder talc, Non genital	1992-	Population-based	2041/2100		6

[53]		use, Genital use (self-report interview)	1997/1998-2002/2003-2008	controls			
Schildkraut et al.2016 [54]	USA	Powder talc<br>Non genital use<br>Genital use (self-report interview)	2010-2015	Population-based controls	584/745	Ed, TL, P, BMI, OC, FH	7.5
Gabriel et al.2019 [55]	USA	Genital talc (self-report interview)	1992-1997/1998-2002/2003-2008	Population-based controls	2040/2100	Men, MS, P, OC, TL, BMI, R, Cont, Smk, AM	6.5
Davis et al.2021 [56]	USA	Genital talc (self-report interview)	2014	Population-based controls	3420/7881	Ed, FH, TL, P, Hy, BMI, Men, Smk	9
*Phung et al.2022 [57]	multi	Any talc exposure, Non genital use, Genital talc (self-report interview)	1992-2010	Population-based controls	8500/13592	R, Ed	8
Leung et al.2022 [58]	Canada	Cosmetic talc (occupational exposure) (self-report interview)	2011–2016	Population-based controls	491/897	Ed, FH, P, MS	7.5
**Endometrial cancer**							
Neill et al, 2012 [64]	Australia	Genital talc (self-report interview)	2005-2007	Population-based controls	1456/749	A, P, OC, HRT, BMI, SMK	8

* Excluded from meta-analysis because of overlap; ** didn't find any case control study for cervical cancer; † Nulliparous women; ‡ In addition to age, calendar year and (in multicenter studies) study center. A, age; P, parity; OC, oral contraceptive; BMI, body mass index; TL, tubal ligation; Smk, tobacco smoking; HRT, hormone replacement therapy; Men, mesopausal status; BF, breast feeding; R, race, Ed, education, FH, family history of ovarian cancer; Hys, hysterectomy; AM, age at menarch; Reg, geographic region; OP, other types of powder; Rel, religion; MS, marital status; Cont, contraceptives other than OC and TL.

Table S5. Newcastle - Ottawa quality assessment scale.

CASE CONTROL STUDIES (maximum score: 9)
**Selection**
**1) Is the case definition adequate?**
a) yes, with independent validation (1)
b) yes, eg record linkage (1) or based on self-reports (0.5)
c) no description (0)
**2) Representativeness of the cases**
a) consecutive or obviously representative series of cases (1)
b) potential for selection biases or not stated (0)
**3) Selection of Controls**
a) community controls (1)
b) hospital controls (0.5)
c) no description (0)
**4) Definition of Controls**
a) no history of disease (endpoint) (1)
b) no description of source (0)
**Comparability**
**1) Comparability of cases and controls on the basis of the design or analysis**
a) study controls for age, gender, province (0)
b) study controls for age, gender, province +smoking (1)
c) study controls for age, gender, province +smoking + other additional factors (2)
**Exposure**
**1) Ascertainment of exposure**
a) secure record (eg surgical records) (1)

b) structured interview where blind to case/control status (1)
c) interview not blinded to case/control status (0.5)
d) written self-report or medical record only (0.5)
e) no description (0)
2) Same method of ascertainment for cases and controls
a) yes (1)
b) no (0)
3) Non-Response rate
a) one or both groups over 90% (1)
b) one or both groups between 60- 90% (0.5)
c) one or both groups under 60% (0)
d) no statemen (0)

COHORT STUDIES (maximum score: 10)

Selection
1) Representativeness of the exposed cohort
a) truly representative of the average _______________ (describe) in the community (2)
b) somewhat representative of the average ______________ in the community (1)
c) selected group of users eg nurses, volunteers (0.5)
d) no description of the derivation of the cohort (0)
2) Selection of the non-exposed cohort
a) drawn from the same community as the exposed cohort (1)
b) drawn from a different source (0.5)
c) no description of the derivation of the non-exposed cohort (0)
3) Ascertainment of exposure
a) secure record (eg surgical records) (1)
b) structured interview (1)
c) written self-report (0.5)
d) no description (0)
4) Demonstration that outcome of interest was not present at start of study
a) yes (1)
b) no (0)

Comparability
1) Comparability of cohorts on the basis of the design or analysis
a) study controls for age, gender, province (0)
b) study controls for age, gender, province +smoking (1)
c) study controls for age, gender, province +smoking + other additional factors (2)

Outcome
1) Assessment of outcome
a) independent blind assessment (1)
b) record linkage (1)
c) self-report (0.5)
d) no description (0)
2) Was follow-up long enough for outcomes to occur
a) yes (select an adequate follow up period for outcome of interest) (1) (average 15 years)
b) no (0)
3) Adequacy of follow up of cohorts
a) complete follow up - all subjects accounted for over 90% (1)
b) subjects lost to follow up unlikely to introduce bias - small number lost - > ____ % (select an adequate %) follow up, or description provided of those lost) between 60- 90% (0.5)
c) follow up rate < ____% (select an adequate %) and no description of those lost under 60% (0)
d) no statemen (0)

Table S6. Relative risk of ovarian cancer by duration of exposure to talc – case-control studies.

Reference	Exposure	Duration category (years)	RR (95% CI)
Chang et al.1997 [38]	After bathing only	<30	1.69 (1.09-2.64)
		30-40	1.43 (0.96-2.15)
		>40	0.86 (0.54-1.38)
Wong et al.1999 [40]	Genital or thigh area	1-9	0.9 (0.6-1.5)
		10-19	1.4 (0.9-2.2)
		>=20	0.9 (0.6-1.2)
Mills et al. 2004 [43]	Perineal talc use	<=3	1.01 (0.58-1.76)
		4-12	1.86 (1.16-2.98)
		13-30	1.45 (0.90-2.32)
		>30	1.22 (0.72-2.08)
Rosenblatt et al. 2011 [49]	Powder after bathing	1-10	1.39 (0.85-2.28)
		10-20	1.46 (0.87-2.45)
		20-35	1.28 (0.78-2.10)
		>35	0.91 (0.51-1.62)
Cramer et al. 2016 [53]	Genital use	<8	1.31 (1.03-1.68)
		8-19	1.31 (1.02-1.68)
		20-35	1.35 (1.07-1.70)
		>35	1.33 (1.03-1.71)
Schildkraut et al. 2016 [54]	Non genital use	<20	1.37 (0.91-2.07)
		>20	1.28 (0.85-1.93)
	Genital use	<20	1.33 (0.95-1.86)
		>20	1.52 (1.11-2.07)
Gabriel et al. 2019 [55]	Genital talc	<=1	1.12 (0.86-1.45)
		1-5	1.37 (1.05-1.77)
		5-24	1.24 (0.97-1.58)
		>25	1.51 (1.17-1.95)
Davis et al. 2021 [56]	Genital talc	<=20	1.43 (1.22-1.68)
		>20	1.28 (1.08-1.51)
Leung et al. 2022 [58]	Cosmetic talc (occupational exposure)	<8	1.68 (0.72-3.93)
		>=8	1.51 (0.36-6.30)

RR, relative risk; CI, confidence interval.

Table S7. Relative risk of ovarian cancer by frequency of exposure to talc in case-control studies.

Reference	Exposure	Original frequency categories	Recorded frequency (times/week)	RR (95% CI)
Whittemore et al.1988 [28]	Any exposure	1-20 times/month	2.45	1.27(0.82-1.96)
		>20 times/month	6.9	1.45(0.94-2.22)
Chang et al. 1997 [38]	After bathing only	<10 times/month	1.16	1.83(1.24-2.73)
		10-25 times/month	4.1	1.12(0.74-1.72)
		>25 times/month	7.6	0.95(0.61-1.49)
Mills et al.2004 [43]	Perineal talc use	rarely or several times/months	0.5	1.34(0.87-2.08)
		1-3 times/week	2	1.16(0.74-1.81)
		4-7 times/week	5.5	1.74(1.14-2.64)
Cramer et al.2016 [53]	Genital use	1-7 days/month	0.8	1.17(0.96-1.44)
		8-29 days/month	4.3	1.37(1.05-1.78)
		>=30 days/month	9.4	1.46(1.2-1.78)

RR, relative risk; CI, confidence interval.

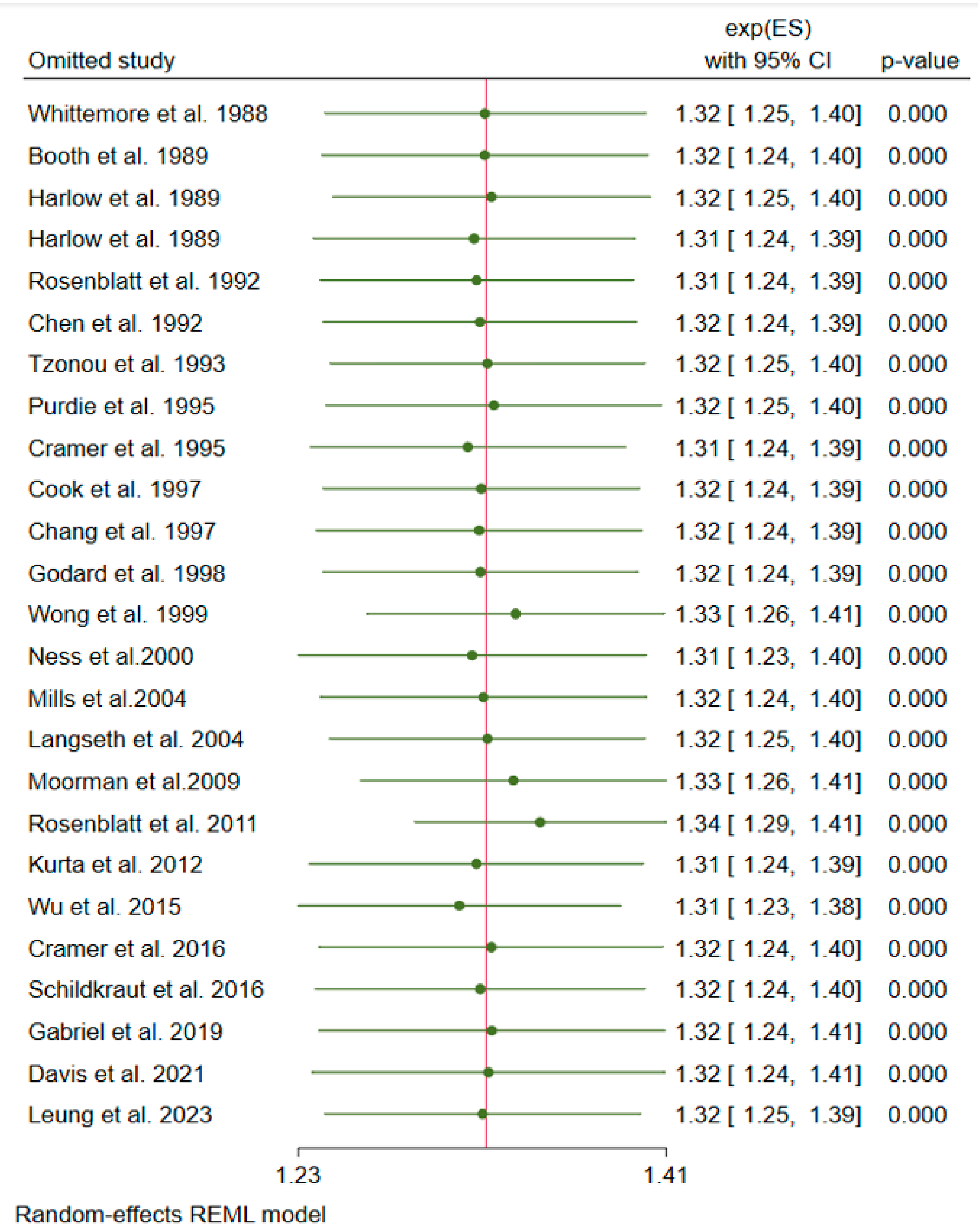

Figure S1. Leave-one-out sensitivity analysis of the association between case control studies related to talc use and ovarian cancer risk.
